# Supplemental parenteral nutrition versus usual care in critically ill adults: a pilot randomized controlled study

**DOI:** 10.1186/s13054-018-1939-7

**Published:** 2018-01-23

**Authors:** Emma J. Ridley, Andrew R. Davies, Rachael Parke, Michael Bailey, Colin McArthur, Lyn Gillanders, D. James Cooper, Shay McGuinness, for the Supplemental Parenteral Nutrition Clinical Investigators, Shay McGuinness, Rachael Parke, Eileen Gilder, Lianne McCarthy, Keri-Anne Cowdrey, Rebecca Baskett, Colin McArthur, Lynette Newby, Lyn Gillanders, Varsha Asrani, Seton Henderson, Jan Mehrtens, Anna Morris, Emmeline Minto, Neil Orford, Allison Bone, Tania Elderkin, Tania Salerno, Roy Hoevenaars, Owen Roodenburg, Meredith Young, Phoebe McCracken, Jasmin Board, Shirley Vallance, Emma Ridley, Eleanor Capel, Paul Young, Leanlove Navarra, Anna Hunt, Sally Hurford, Lynn Andrews, Diane Mackle, Catherine Boulton, Michael Bailey, Andrew Davies, Adam Deane, Carol Hodgson, Emma Ridley

**Affiliations:** 1 Australian and New Zealand Intensive Care Research Centre, School of Public Health and Preventative Medicine, Monash University, Level 3, 553 St Kilda Road, Melbourne, 3004 Australia; 2 Nutrition Department, Alfred Health, Commercial Road, Melbourne, VIC 3004 Australia; 3 Cardiothoracic and Vascular Intensive Care Unit, Auckland City Hospital, Park Road, Grafton, Auckland, New Zealand; 4 Intensive Care Unit, The Alfred Hospital, Commercial Road, Melbourne, VIC 3004 Australia; 5 Medical Research Institute of New Zealand, Wellington, New Zealand; 6 The Department of Critical Care Medicine, Auckland City Hospital, Park Road, Grafton, Auckland, New Zealand; 7 Nutrition and Dietetics, Auckland City Hospital, Park Road, Grafton, Auckland, New Zealand; 8 Faculty of Medical and Health Sciences, University of Auckland, Park Road, Grafton, Auckland, New Zealand

**Keywords:** Enteral nutrition, Parenteral nutrition, Randomized controlled trial, Nutrition therapy, Clinical nutrition, Critical care, Intensive care

## Abstract

**Background:**

In the critically ill, energy delivery from enteral nutrition (EN) is often less than the estimated energy requirement. Parenteral nutrition (PN) as a supplement to EN may increase energy delivery. We aimed to determine if an individually titrated supplemental PN strategy commenced 48–72 hours following ICU admission and continued for up to 7 days would increase energy delivery to critically ill adults compared to usual care EN delivery.

**Methods:**

This study was a prospective, parallel group, phase II pilot trial conducted in six intensive care units in Australia and New Zealand. Mechanically ventilated adults with at least one organ failure and EN delivery below 80% of estimated energy requirement in the previous 24 hours received either a supplemental PN strategy (intervention group) or usual care EN delivery. EN in the usual care group could be supplemented with PN if EN remained insufficient after usual methods to optimise delivery were attempted.

**Results:**

There were 100 patients included in the study and 99 analysed. Overall, 71% of the study population were male, with a mean (SD) age of 59 (17) years, Acute Physiology and Chronic Health Evaluation II score of 18.2 (6.7) and body mass index of 29.6 (5.8) kg/m^2^. Significantly greater energy (mean (SD) 1712 (511) calories vs. 1130 (601) calories, *p* < 0.0001) and proportion of estimated energy requirement (mean (SD) 83 (25) % vs. 53 (29) %, *p* < 0.0001) from EN and/or PN was delivered to the intervention group compared to usual care. Delivery of protein and proportion of estimated protein requirements were also greater in the intervention group (mean (SD) 86 (25) g, 86 (23) %) compared to usual care (mean (SD) 53 (29) g, 51 (25) %, *p* < 0.0001). Antibiotic use, ICU and hospital length of stay, mortality and functional outcomes were similar between the two groups.

**Conclusions:**

This individually titrated supplemental PN strategy applied over 7 days significantly increased energy delivery when compared to usual care delivery. Clinical and functional outcomes were similar between the two patient groups.

**Trial registration:**

Clinical Trial registry details: NCT01847534 (First registered 22 April 2013, last updated 31 July 2016)

**Electronic supplementary material:**

The online version of this article (10.1186/s13054-018-1939-7) contains supplementary material, which is available to authorized users.

## Background

Best practice guidelines for energy delivery in critical illness often recommend that energy delivery be aimed to meet energy requirements, usually estimated using standard equations, and most often using enteral nutrition (EN) [1–4]. However, energy delivery in critically ill patients when using EN alone is almost always less than estimated requirements [5, 6]. Parenteral nutrition (PN), delivered in addition to EN, is a strategy which may increase energy delivery more closely to estimated energy requirements, however recommendations for use differ and evidence is controversial [1–4, 7–12]. Previously, the use and infective risk of PN has been a concern when compared to standard care nutrition, however, this has been challenged in more recent trials which investigated PN in a modern-day ICU setting [13, 14].

Observational studies have suggested an association between higher energy delivery and improved clinical outcomes. [15–18]. And, prospective randomized controlled trials (RCTs) addressing this question have been limited by either trial size, or by methodological concerns [19]. One randomized trial found that supplemental PN was associated with decreased infective complications later in ICU stay (however this endpoint was not in the original study protocol), and another found a trend to improved outcomes in nutritionally at-risk patients [8, 11]. The largest randomized trial indicated harm with early supplemental PN delivery, despite only achieving 74% of estimated energy requirements in the early PN arm [7, 10]. Further, interpretation of this trial was complicated by the parallel use of an intensive insulin therapy strategy, which has since been found to impair patient outcomes [20].

We aimed to determine if an individually titrated supplemental PN strategy commenced 48–72 hours following ICU admission and continued for up to 7 days would increase energy delivery closer to estimated requirements in critically ill adults compared to usual care delivery. Secondary aims (which are not reported in this article) were to determine rates of enrolment, feasibility of trial processes and estimate sample size to assist planning a large randomized trial.

## Methods

### Design

We conducted a prospective, unblinded, parallel group, block randomized phase II pilot trial in six ICUs in Australia and New Zealand.

### Patients

Patients aged ≥ 16 years, admitted to ICU in the previous 48–72 hours, who were receiving mechanical ventilation (MV) and expected to continue until the day after randomization, with central venous access and one or more defined organ system failure were eligible. Patients were excluded if they could not receive EN and/or PN at the time of randomization, were already receiving PN, had a requirement for a specific PN solution (e.g. glutamine containing), had received more than 80% of their estimated nutrition requirements from EN in the 24 hours prior to randomization, seemed not likely to survive the subsequent 96 hours, had a treatment limitation in place or a high likelihood of terminal illness, were pregnant or the treating clinician did not believe that study participation was in the best interests of the patient. There was a modification to the inclusion criteria after the first 6 months of recruitment. Details of the full inclusion and exclusion criteria can be viewed in Additional file 1.

Eligible patients were randomly assigned in a 1:1 ratio via a web-based randomization system. Randomization was stratified by site and allocation occurred in permuted blocks of two, four or six. Recruitment began on 17 February 2014 and was completed on 6 January 2016 with the final outcome determined 180 days later. Ethics approval was obtained from The Alfred Hospital Research and Ethics committee for Australia and the Northern A Health and Disability Ethics Committee in New Zealand, as well as the Monash University Research and Ethics Committee. As participants were unable to provide consent for participation at the time of enrolment, the patient’s legal surrogate, relative/friend or whanau member was approached for consent or agreement to participate in the study. Patients were approached at a later time if it was appropriate and they regained the capacity to provide consent to continue to participate. The full protocol for this RCT was pre-published and registered (NCT01847534) [21].

### Study processes

#### Common to both groups

Body weight was standardized in both groups using ‘calculated body weight’ (CBW) as follows:

Body mass index (BMI) < 25 kg/m^2^: actual body weight was equal to CBW BMI ≥ 25 kg/m^2^: CBW was an ideal body weight set at a body mass index (BMI) of 23 kg/m^2^ using the patient’s height.

Actual body weight was preferred to estimated weight if it was current within 6 weeks and height was estimated using demi-arm span [22]. Once set, the CBW was not changed for the duration of the study.

Energy requirements were determined on a daily basis using a fixed prescription method of 25 kilocalories (kcal)/kg CBW or 30 kcal/kg CBW if the patient was receiving renal replacement therapy or extracorporeal membrane oxygenation on that day. The daily nutrition target was 100% of estimated energy requirement in both groups. Estimated protein requirements and the choice of EN formula followed usual practice at the participating ICU and recorded as part of study data collection. To determine the volume of EN received over 24 hours, discarded gastric residual volumes were deducted from the total volume of EN received. Blood glucose level (BGL) management followed the participating ICUs usual practice, which was usually based on the control group strategy in a recently conducted trial [20].

#### Management of the usual care group

Nutrition therapy in the usual care group followed clinical practice at the participating ICU. PN was only used when EN delivery remained insufficient despite attempts to improve it with strategies recommended in best practice guidelines [1, 3, 4]. If PN was required during the first 7 days of the study in the usual care group, the same PN formulation used in the intervention group was provided. If PN was required in usual care after the first 7 days the usual hospital PN formulation was used. Micronutrients were provided as part of the standard EN solutions provided in usual care. Additional micronutrients could be provided if deemed necessary by the treating clinical team.

#### Management of the intervention group

**Fig. 1:**
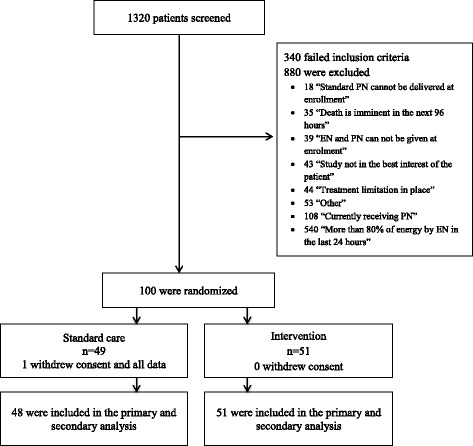
Participant flow diagram

The intervention group received a supplemental PN strategy, delivered for up to 7 days after randomization, using Olimel N9-840/Triomel with added multi-trace elements and multi-vitamins (manufactured and supplied by Baxter Healthcare Corporation, Sydney, Australia). On randomization, intervention PN was commenced within 2 hours, at a rate based on the percentage of estimated energy requirements received from EN in the 24 hours prior to randomization. These rates corresponded to either 40% or 80% of the estimated requirement (Fig. 1, panel A in Additional file 1 demonstrates study processes at randomization). The intervention strategy was designed to increase average delivery towards 100% of the estimated energy requirement but avoid overfeeding by (1) using ideal body weight in those who were overweight or obese, (2) having variable PN rates which were individually titrated, reviewed daily and based on the percentage estimated energy requirement delivered, (3) accounting for additional energy from EN, intravenous glucose solutions ≥ 25% and propofol and (4) never providing more than 80% of the estimated energy requirement by the intervention PN.

After the day of randomization, total energy received from EN, propofol and intravenous glucose solutions ≥ 25% were assessed daily for up to 7 days by a dietitian, research coordinator or investigator at the site. Based on the percentage of estimated energy requirement received, the intervention PN was individually titrated on a daily basis, with three rates possible for the following 24 hours (corresponding to 0%, 40% or 80% of estimated requirements). Once set, the PN was continued at that rate for the next 24 hours and EN was managed as per standard practice in the participating ICU and not reduced based on the intervention strategy. If there was a discontinuation of EN ≥ 2 hours, the intervention PN was run at a rate corresponding to 80% of the estimated energy requirement for the duration of the interruption and once EN was recommenced, returned to the last rate determined. The intervention period ceased at the end of study day 7 or earlier if the patient was discharged from the ICU or oral nutrition was commenced.

Details on the interventional product and daily management of intervention PN can be viewed in Additional file 1 (Table 1 and Fig. 1, panel B).

**Table 1:** Baseline characteristics

Variable	Usual care (*n* = 48)	Intervention (*n* = 51)
Age, years, mean (SD)	60 (17)	59 (17)
Sex, male, n (%)	35 (73)	35 (69)
BMI, kg/m^2^, mean (SD)	30 (6)	29 (6)
APACHE II score, mean (SD)	19 (7)	18 (7)
APACHE III diagnosis code, n (%)		
Cardiovascular	29 (59)	31 (61)
Trauma	7 (14)	6 (12)
Respiratory	6 (12)	3 (6)
Sepsis	1 (2)	7 (14)
Gastrointestinal	1 (2)	2 (4)
Musculoskeletal	2 (4)	0 (0)
Renal	1 (2)	1 (2)
Unknown	1 (2)	1 (2)
Neurological	1 (2)	0 (0)
Location prior to ICU admission, n (%)		
Elective surgery	20 (42)	22 (43)
ICU	9 (19)	7 (14)
Emergency surgery	9 (19)	5 (10)
ED	5 (10)	8 (16)
Ward	4 (8)	6 (12)
Other hospital	1 (2)	3 (6)
Time from hospital admission to randomization, days, median [IQR]	3 [3–6]	3 [3–4]
Time from ICU admission to randomization, days, mean (SD)	2.5 (0.4)	2.5 (0.4)
Baseline total SOFA, mean (SD)	10 (3)	10 (4)
Bloods, median [IQR]		
ALT, U/L	25 [11–103]	40 [18–108]
ALP, U/L	67 [49–97]	72 [50–89]
GGT, U/L, mean (SD)	44 [27–79]	41 [28–98]
Bilirubin, mmol/L	21 [10–41]	24 [11–47]
WCC, 0^9/L	13 [10–15]	17 [11–23]
TG, mmol/L	2 [1–3]	2 [1–3]
CRP, mg/L	209 (97)	217 (111)
Mid arm muscle circumference, cm, mean (SD)	34 (5)	34 (4)

*APACHE* Acute Physiology and Chronic Health Evaluation II, *ALP* alkaline phosphatase, *ALT* alanine aminotransferase, *BMI* body mass index, *CRP* C-reactive protein, *ED* Emergency department, *GGT* gamma-glutamyltransferase, *ICU* intensive care unit, *IQR* interquartile range, *SD* standard deviation, *SOFA* Sequential Organ Failure Assessment, *TG* triglyceride, *WCC* white cell count

### Data collection

Baseline data included nutrition information, patient and ICU admission demographics, severity of illness characteristics and standard blood test results. Daily data included nutrition requirements and intake (including energy from propofol and intravenous glucose solutions ≥ 25%); morning BGL level; number of episodes of hypoglycaemia; complications associated with nutrition delivery and antibiotic usage. On specific days Sequential Organ Failure Assessment (SOFA) score, liver function tests, white cell count, serum triglyceride, and C-reactive protein were collected.

### Outcomes

The primary outcome was mean energy delivered from both EN and/or PN therapy through the first 7 days of the study (the intervention period). Secondary outcomes included: (1) total protein delivered in the first 7 days of the study period; (2) total energy and protein delivered in the ICU stay (up to 28 days); (3) number of new antibiotics commenced while in ICU to day 28; (4) SOFA scores; (5) duration of MV to day 28; (6) duration of ICU and hospital stay; (7) mortality to 180 days post randomization; (8) assessment of physical function using the ICU mobility scale (or 6-minute walk test where possible) at hospital discharge (D/C), hand grip strength (HGS) at ICU and hospital D/C and (9) quality of life with the EuroQuol-5 Dimension 3 Level (EQ-5D-3L) at hospital D/C, 90 and 180 days post randomization [23, 24].

### Statistical analysis

A sample size of 100 patients was calculated on a mean (SD) daily delivery of 1400 (600) calories in the usual care group, estimated from work previously conducted by our group [25, 26]. This provided an 80% power (two-sided *p* value of 0.05) to detect a 30% relative increase (1400 vs. 1820 kcal) in calories delivered.

Daily data were collected until the patient was discharged from ICU, died or was censored at day 28 (whichever occurred first). We conducted all analyses according to the intention-to-treat principle and there were no planned interim analyses. Baseline and outcome variables were compared using chi-square tests for equal proportion, Student’s *t* test for normally distributed outcomes and Wilcoxon rank-sum tests otherwise with results reported as numbers (percentages), means (SD) or medians [interquartile range (IQR)] respectively. Longitudinal analysis of total energy was performed using mixed linear modelling with patients treated as random effects, fitting main effect for treatment and time and an interaction between the two to determine if treatment behaved differently over time. Missing data were not imputed and no assumptions were made relating to missingness. All analysis was performed using SAS version 9.4 (SAS Institute Inc., Cary, NC, USA) and a two-sided *p* value of 0.05 was considered to be statistically significant.

## Results

### Patients

Of 1320 patients screened for eligibility, 100 patients were randomized over 24 months. One patient in the usual care group withdrew consent for follow-up and use of all data (Fig. 1).

### Baseline characteristics

The two groups were comparable at baseline (Table 1). The mean (SD) age was 59 (17) years, 71% were male and the mean Acute Physiology and Chronic Health Evaluation II score was 18.2 (6.7). Fewer patients with a diagnostic category of ‘sepsis’ were randomized to usual care than the intervention group (one and seven patients respectively). Prior to randomization, more patients in the usual care group (44 (91%)) had commenced EN compared to the intervention group (40 (78%)). The median [IQR] energy received in the usual care group was less (394 [67–1020] kcal) than the intervention group (605 [75–1270] kcal) prior to randomization. The mean overall estimated energy and protein requirements in both groups at randomization were 2092 (392) kcal and 103 (21) g.

### Nutrition delivery

**Fig. 2:**
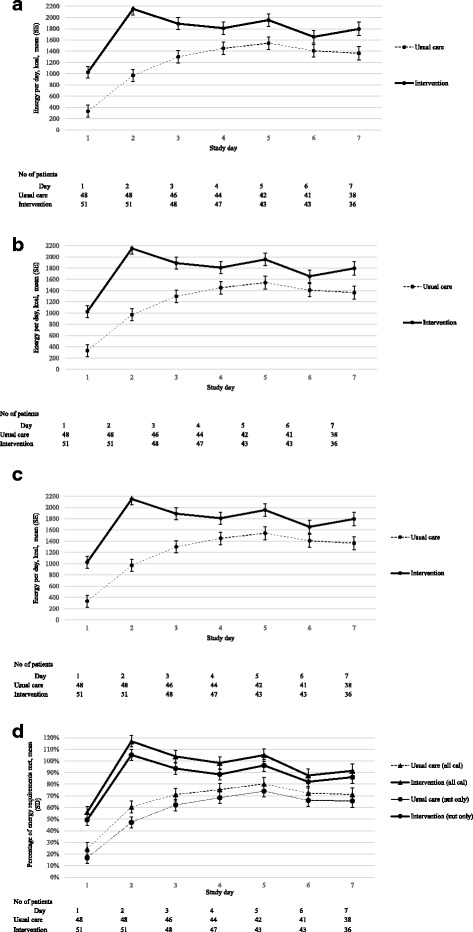
Daily energy and protein intake during the 7-day intervention period. Mean standard error (SE) daily energy and protein intake during the 7-day intervention period: Panel (**a**) Energy from nutrition only (kcal); Panel (**b**) energy from all sources (kcal); Panel (**c**) protein from nutrition (g); Panel (**d**) percentage of estimated energy requirements received from nutrition and all sources

The median time from randomization to commencing the intervention was 1.2 [0.5–1.8] hours. Over the 7-day intervention period, the mean daily energy delivery from EN, PN or both in usual care was 1130 (601) kcal and 1712 (511) kcal in the intervention group, *p* = < 0.0001. When energy from nutrition, propofol and intravenous glucose solutions ≥ 25% were included the mean daily intake increased to 1298 (671) kcal in the usual care group and 1892 (540) kcal in the intervention group, *p* < 0.0001. Those in the usual care group were delivered a mean 53 (29) g of protein daily compared to 86 (35) g of protein daily in the intervention group, *p* < 0.0001. Figure 2, panels A, B and C demonstrate energy and protein intake on a daily basis over the 7-day intervention period. On study day 2, those in the intervention group received a mean proportion of estimated energy requirement of 105% (5%) and when energy from all sources were accounted for this increased to 117% (5%) (Fig. 2, Panel D). On all other study days, the proportion of estimated energy requirement provided was less than 100%. Figure 2 in the Additional file 1 shows the proportion of daily energy delivery by EN and PN in the usual care (Panel A) and the intervention (Panel B) groups. Over the duration of ICU stay, mean energy and protein from nutrition were 1212 (676) kcal and 57 (33) g protein in the usual care group compared to 1599 (458) kcal and 79 (23) g protein in the intervention group, (*p* = 0.001 and < 0.0001, respectively). Including all energy sources for the duration of ICU stay increased the mean energy to 1331 (720) kcal and 1718 (468) kcal in the usual and intervention groups, respectively, *p* < 0.0001. Table 2 provides further information about energy delivery during the intervention period and ICU stay. There were ten patients in the usual care group who received PN during the intervention period; the median time to commencement was 3 [1–4] days.

**Table 2:** Energy and protein delivery during the 7-day intervention and ICU stay

Variable	Usual care (*n* = 48)	Intervention (*n* = 51)	*p* value
7-day intervention period, mean (SD)			
Delivery of energy from EN and PN, kcal	1130 (601)	1712 (511)	<0.0001
Proportion of energy from EN and PN, %	54 (28)	83 (22)	<0.0001
Energy from EN and PN, kcal/kg	13 (6.6)	20.6 (6.3)	<0.0001
Delivery of energy from all sources, kcal	1298 (671)	1892 (540)	<0.0001
Proportion of energy from all sources, %	62 (31)	92 (22)	<0.0001
Energy from all sources, kcal/kg	16.8 (8.2)	24.9 (6.4)	<0.0001
Delivery of protein, g	53.3 (28.5)	85.6 (25.4)	<0.0001
Proportion of protein, %	51 (25)	86 (23)	<0.0001
Protein delivery, g/kg	0.6 (0.3)	1.0 (0.3)	<0.0001
ICU stay, mean (SD)			
Delivery of energy from EN and PN, kcal	1212 (676)	1599 (458)	0.001
Proportion of energy from EN and PN, %	58 (30)	78 (21)	<0.0001
Energy from EN and PN, kcal/kg	13.9 (7.4)	19.2 (5.7)	<0.0001
Delivery of energy from all sources, kcal	1331 (720)	1718 (468)	0.002
Proportion of energy from all sources, %	63 (32)	84 (21)	<0.0001
Energy from all sources, kcal/kg	15.3 (7.8)	20.6 (5.7)	<0.0001
Delivery of protein, g	57 (33)	79 (23)	<0.0001
Proportion of protein, %	54 (29)	80 (22)	<0.0001
Protein delivery, g/kg	0.7 (0.3)	1.0 (0.3)	<0.0001

*EN* enteral nutrition, *PN* parenteral nutrition; *kcal* kilocalorie, *SD* standard deviation

### Other outcomes

Morning BGL was lower in the usual care (mean 7.9 (1.9) mmol/L) compared to the intervention (8.5 (1.2) mmol/L, *p* = 0.03) group, as was daily insulin dose (median 8 [0–35] compared to 24 [4–69] units in the usual care and intervention groups, respectively, *p* = 0.03). There were 16 (33%) and 18 (35%) patients in the usual care and intervention groups, respectively, who received at least one new antibiotic during the study period, *p* = 0.84. There were no significant differences between the two groups in the duration of mechanical ventilation, ICU or hospital stay, mortality, witnessed complications of feeding or functional outcomes (Table 3).

**Table 3:** Clinical outcomes

Variable	n	Usual care	n	Intervention	*p* value
Patients with reported complications during study period, n (%)					
GRV > 300 ml on study days 1–7	48	23 (48)	51	28 (55)	0.49
Abdominal distention		14 (29)		16 (31)	0.81
Vomiting		8 (17)		13 (26)	0.28
Calories from propofol over the study period, kcal, median [IQR]	48	0 [0-110]	51	0 [0-160]	0.48
Blood test results on study day 7:					
ALP, U/L, mean (SD)	38	165 (81)	33	183 (103)	0.40
ALT, U/L, median [IQR]	38	50 [23-86]	34	58 [30-102]	0.54
GGT, U/L, mean (SD)	38	196 (125)	34	216 (126)	0.51
Bilirubin, mmol/L, median [IQR]	38	15 [11-29]	35	24 [14-53]	0.47
WCC, 0^9/L, mean (SD)	38	18 (10)	36	20 (10)	0.18
TG, mmol/L, median [IQR]	37	2 [1-3]	32	2 [2-4]	0.18
CRP, mg/L, median [IQR]	37	110 [78-185]	32	153 [105-216]	0.06
Mean SOFA over study duration, mean (SE)	48	8.0 (0.4)	51	8.2 (0.4)	0.75
Duration of mechanical ventilation, days, median [IQR]	48	8 [5-18]	51	10 [6-15]	0.68
Duration of ICU stay, days, median [IQR]	48	11 [6-17]	51	11 [5-17]	0.83
Duration of hospital stay, days, mean (SD)	48	23 (17)	51	22 (21)	0.85
Survival					
ICU D/C, n (%)		37 (77)		36 (71)	0.46
Hospital D/C, n (%)	48	37 (77)	51	35 (67)	0.37
90 days, n (%)		35 (73)		32 (63)	0.28
180 days, n (%)		35 (73)		32 (63)	0.28
EQ-5D-3L					
Hospital D/C, mean (SD)	17	0.32 (0.36)	27	0.25 (0.34)	0.54
90 days, median [IQR]	29	0.76 (0.23)	35	0.69 (0.24)	0.29
180 days, mean (SD)	29	0.77 (0.24)	35	0.75 (0.26)	0.76
Hand grip strength at hospital D/C, kg, mean (SD)	24	20 (8)	19	19 (13.5)	0.71
ICU mobility scale at hospital D/C, median [IQR]	33	8 [4-10]	25	9 [5-10]	0.58
Mid arm muscle circumference, hospital D/C, cm, mean (SD)	25	30 (5)	22	30 (5)	0.91

The highest level of function scale ranges from 0 to 10 with 0 being ‘no mobility’ (lying in bed) and 10 being ‘Walking independently without a gait aid’ [23].

*ALT* alanine aminotransferase, *ALP* alkaline phosphatase, *BMI* body mass index, *CRP* C-reactive protein, *D/C* discharge, *ED* Emergency department, *EQ-5D-3L* EuroQuol-5 Dimension 3 Level, *GGT* gamma glutamyltransferase, *GRV* gastric residual volume, *ICU* intensive care unit, *IQR* interquartile range, *SD* Standard deviation, *SOFA* Sequential Organ Failure Assessment, *TG* triglyceride, *WCC* white cell count

## Discussion

### Key findings

Our multicentre, pilot, randomized trial in 100 critically ill adults receiving EN, found that an individually titrated supplemental PN strategy was feasible and effective in delivering increased energy, closer to estimated requirements than usual care. There were no differences between our two groups in any clinical outcomes.

Previous studies have found that use of supplemental PN can deliver additional energy in critical illness when combined with EN [8, 11, 27, 28]. However, the largest randomized trial addressing this question, achieved no more than approximately 74% of estimated energy requirements [7]. Our trial found that a supplemental PN strategy could instead be used to increase energy delivery closer to the patient’s estimated energy requirement and includes several different approaches to help protect against overfeeding, an essential element of any supplemental PN intervention.

Despite many interventions aiming to improve energy delivery, the timing, and the amount of energy to provide in critical illness remains uncertain. Recently, a U-shaped relationship between energy needs and clinical outcomes has been suggested, with just 70% of the measured requirement being optimal for patient outcomes in a cohort trial [29]. It has been suggested that increased macronutrient delivery early in ICU admission may be harmful by inhibiting autophagy, an important and protective cell process for maintenance of organ function [7, 30]. These factors may explain indications of harm in patients who received early supplemental PN (74% of energy requirement) compared to those who received late PN (30% of energy requirement) in a large RCT [7]. Furthermore, a recent randomized trial found no advantage from increasing energy delivery using PN to requirements guided by indirect calorimetry during the first week of critical illness, although the trial was likely to be underpowered for clinical outcomes [28]. And a recent meta-analysis suggested higher infectious complications in a sub-group of studies where patients received considerably more energy from PN compared to EN alone [31].

It is also possible that energy requirements during critical illness vary during the time course of critical illness. Early in ICU admission, endogenous glucose supplies are mobilised (up to 1500 kcal/day) and metabolic rate reduces as a result of the metabolic response to illness [32]. Less energy from exogenous sources may then be required early in critical illness, and this may explain why studies of short duration hypocaloric nutrition, early in illness, have suggested equivalence to usual care [7, 33, 34]. We found no indicators of overfeeding in our trial but indirect calorimetry was not used. Later in the time course of critical illness, energy requirements may change and increase as a patient’s metabolism switches from a catabolic to anabolic state. It is plausible that provision of nutrition in this anabolic phase may be more important than in the early phase. These factors may partially explain why nutrition trials, which have predominately investigated the early phase of illness, have been unable to demonstrate patient benefit to date. Furthermore, the use of predictive equations to estimate energy expenditure during critical illness is known to be inaccurate when compared to indirect calorimetry [35–37]. Use of indirect calorimetry to guide energy delivery may result in improved clinical and functional outcomes; however, this remains to be determined in future prospective controlled trials.

Despite these concerns, many observational studies have suggested higher energy delivery is positively associated with improved clinical outcomes [15–18, 38]. And, even in the absence of randomized trial data in support, some best practice guidelines recommend the delivery of energy to approximate estimated energy requirements [1–4]. The recommendations from best practice nutrition guidelines need to be interpreted carefully however; some have not been updated in recent years (when critical care nutrition research has been prolific), and all are developed with different methodologies. Both of these factors complicate comparisons and interpretation of the evidence [1–4, 12].

### Strengths and limitations

Our usual care patients received energy delivery comparable with current clinical practice as reported in recent cohort studies and multiple approaches to reduce the risk of overfeeding were used [5, 6]. We did observe a significantly higher dose of insulin in our intervention group, which could simply reflect the increased dextrose load or which instead could be an early indication of overfeeding. Rates of hypoglycemia were not different between our groups. On only 1 of 7 intervention days, was energy delivery greater than the estimated requirements (117% of estimated energy requirements on day 3) and the effect of this single day on overall trial outcomes cannot be determined. After study day 3, while still remaining statistically significant, the energy difference between our two groups was relatively small at approximately 200 kcal/day. Though this did remain statistically significant this relatively small difference may not be clinically significant. Our trial was designed as a feasibility study, has small patient numbers, and therefore was not powered to detect differences in clinical outcomes. Our significant proportion of cardiovascular patients may also limit generalizability. We used ‘administration of new antibiotics’ as a surrogate marker for development of infective complications; however, the safety of PN when applied in a modern ICU setting has recently been challenged in two large RCTs [13, 14]. Our loss to follow-up for our functional secondary outcomes measured at ICU and hospital discharge was also significant as patients were often unable to participate in the assessments. Finally, data collection on nutrition intake ceased when oral intake commenced in ICU, however the contribution of this oral intake to overall energy balance is likely to be small and balanced between the two groups.

## Conclusions

Our individually titrated supplemental PN strategy was feasible and effective at increasing energy delivery closer to estimated requirements in critically ill adults. To determine the impact of this strategy on patient outcomes, or to determine the optimal timing for such a strategy during the changing time course of critical illness would require substantially larger, carefully timed, randomized trials.

## Additional file

Additional file 1: Supplemental parenteral nutrition versus usual care in critically ill adults: a pilot randomized controlled study. (DOCX 94 kb)

## Abbreviations

APACHE: Acute Physiology and Chronic Health Evaluation II
BGL: Blood glucose level
CBW: Calculated body weight
D/C: Discharge
ED: Emergency department
EN: Enteral nutrition
EQ-5D-3L: EuroQuol-5 Dimension 3 Level
ICU: Intensive care unit
kcal: Kilocalorie
LOS: Length of stay
MV: Mechanical ventilation
PN: Parenteral nutrition
RCT: Randomized controlled trial
SOFA: Sequential Organ Failure Assessment
