# Supplementary material for: Supplemental parenteral nutrition versus usual care in critically ill adults: a pilot randomized controlled study
Source: Crit Care. 2018 Jan 23;22:12. doi: 10.1186/s13054-018-1939-7 (PMC5781264; doi:10.1186/s13054-018-1939-7)
Supplement: Additional file 1: — Supplemental parenteral nutrition versus usual care in critically ill adults: a pilot randomized controlled study. (DOCX 94 kb) [file 13054_2018_1939_MOESM1_ESM.docx]

**ADDITIONAL FILE**

**Supplemental parenteral nutrition in critically ill adults: a pilot randomized controlled study**

Emma J Ridley^1,2^, Andrew R Davies^1^, Rachael Parke^1,3,5,8^, Michael Bailey^1^, Colin McArthur^6^, Lyn Gillanders^6, 7, 8^, D James Cooper^1, 4^, Shay McGuinness ^1,3,5^ for the Supplemental Parenteral Nutrition Clinical Investigators

^1^ Australian and New Zealand Intensive Care Research Centre, School of Public Health and Preventative Medicine, Monash University, Commercial Road, Melbourne, 3004, Australia

^2^ Nutrition Department, Alfred Health, Commercial Road, Melbourne, 3004, Australia

^3^ Cardiothoracic and Vascular Intensive Care Unit, Auckland City Hospital, Park Road, Grafton, Auckland New Zealand

^4^ Intensive Care Unit, The Alfred Hospital, Commercial Road, Melbourne, 3004, Australia

^5^ Medical Research Institute of New Zealand, Wellington, New Zealand

^6^ The Department of Critical Care Medicine, Auckland City Hospital, Park Road, Grafton, Auckland, New Zealand

^7^ Nutrition and Dietetics, Auckland City Hospital, Park Road, Grafton, Auckland New Zealand

^8^ Faculty of Medical and Health Sciences, University of Auckland, Park Road, Grafton, Auckland, New Zealand

**Contents**

[1. Inclusion and exclusion criteria 3](#_Toc498325888)

[2. Figure 1: Study processes in the intervention arm 5](#_Toc498325890)

[3. Table 1: Product information for Olimel N9-840E/Triomel 9 with electrolytes and additions 6](#_Toc498325891)

[4. Figure 2: Mean proportion of daily energy intake provided by enteral and parenteral nutrition during the 7 day intervention period 7](#_Toc498325892)

[5. Protocol deviations during study period 9](#_Toc498325894)

[6. Adverse events 9](#_Toc498325895)

# Inclusion and exclusion criteria

Inclusion criteria

Patients in intensive care who meet all of the following:

- Admitted to intensive care between 48 hours and 72 hours previously
- Mechanically ventilated at the time of enrolment and expected to remain ventilated until the day after tomorrow
- At least 16 years of age
- Have central venous access suitable for parenteral nutrition (PN) solution administration
- Have 1 or more organ system failure (respiratory, cardiovascular or renal) related to their acute illness defined as:

1. PaO_2_/FiO_2_ ≤ 300 mmHg^*^
2. Currently on 1 or more continuous vasopressor infusion which were started at least 4 hours ago at a minimum dose of :
   1. Dopamine greater than 5 mcg/kg/min
   2. Noradrenaline ≥ 0.1mcg/kg/min
   3. Adrenaline ≥ 0.1 mcg/kg/min
   4. Any dose of total vasopressin
   5. Milrinone >0.25mcg/kg/min)
3. Renal dysfunction defined as

In patients without known renal disease:

- 1. Serum Creatinine > 171 mmol/l OR
  2. Currently receiving renal replacement therapy

In patients with known renal disease:

1. an absolute increase of > 50% in serum Creatinine from baseline OR
2. Currently receiving renal replacement therapy
3. Currently has an intracranial pressure monitor or ventricular drain in situ^+^
4. Currently receiving extracorporeal membrane oxygenation^+^
5. Currently has a ventricular assist device^+^

### Exclusion criteria

Patients will be excluded if:

- Both enteral nutrition (EN) and PN cannot be delivered at enrolment (i.e. either an enteral tube or a central venous catheter cannot be placed or clinicians feel that EN or PN cannot be safely administered due to any other reason).
- Currently receiving PN
- Standard PN solutions cannot be delivered at enrolment (i.e. clinicians believe that a patient definitely needs a specific parenteral nutrition formulation (e.g. glutamine-supplementation or specific lipid formulation).
- Death is imminent or deemed highly likely in the next 96 hours.
- There is a current treatment limitation in place or the patient is unlikely to survive to 6 months due to underlying illness
- More than 80% of energy requirements have been satisfactorily delivered via the enteral route in the last 24 hours.
- Are known to be pregnant
- The treating clinician does not believe the study to be in the best interest of the patient

^*^Modified from PaO_2_/FiO_2_ ≤ 200 mmHg during protocol amendment, after recruitment commenced

^+^Added during protocol amendment, after recruitment commenced

# Figure 1: Study processes in the intervention arm

Panel A) Study processes at randomization; Panel B) Daily adjustment of intervention


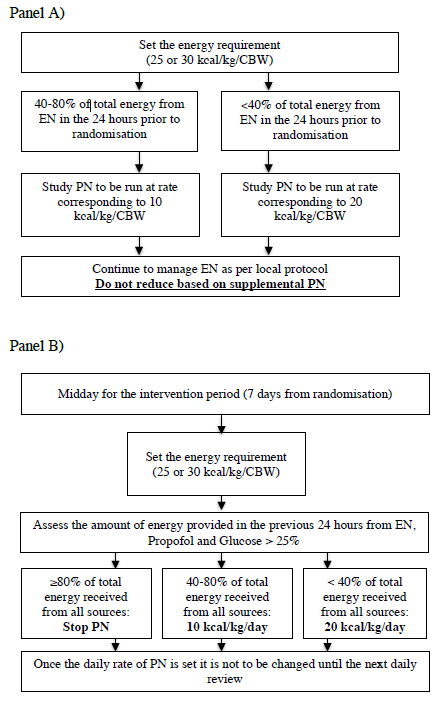


CBW: Calculated body weight; EN: Enteral *nutrition*; PN: Parenteral nutrition; kcal: Kilocalorie

# Table 1: Product information for Olimel N9-840E/Triomel 9 with electrolytes and additions

| **Contents** | **Compounded Ready To Use Parenteral Nutrition (per 1500ml bag)** |
| --- | --- |
| Total nitrogen (g) | 13.5 |
| Amino acid (g) | 85.4 |
| Glucose (g) (Hydrous) | 181.5  (equal to Anhydrous 165 g labelled on compounded bag) |
| Lipid as ClinOleic (g) | 60 |
| Total energy (kcal) | 1600 |
| Non protein energy (kcal) | 1260 |
| Glucose energy (kcal) | 660 |
| Lipid energy (kcal) | 600 |
| Sodium (mmol) | 52.5 (New Zealand)  54 (Australia - includes 1.5 mmol from Sodium Ascorbate) |
| Potassium (mmol) | 45 |
| Magnesium (mmol) | 6.0 |
| Calcium (mmol) | 5.3 |
| Phosphate (mmol) | 22.5 |
| Acetate (mmol) | 80 |
| Chloride (mmol) | 68 |
| Osmolarity (mOsm/L) | 1310 |
| **Additions per bag of parenteral nutrition** | |
| Baxter’s Multiple Trace Elements with Iron (mcg) | Per ml (note 10ml is added to each parenteral nutrition bag) |
| Zinc | 650 |
| Copper | 130 |
| Manganese | 27 |
| Chromium | 1 |
| Selenium | 3.2 |
| Iodide | 13 |
| Molybdenum | 1.9 |
| Iron | 120 |
| Ascorbate (Vitamin C) for stability (mg per bag)  Sodium Ascorbate in Australia and Ascorbate acid in NZ | 300 |
| Cernevit (ml per bag) | 5 |

# Figure 2: Mean proportion of daily energy intake provided by enteral and parenteral nutrition during the 7 day intervention period

Panel A) Components of total energy intake from EN, PN and non-study PN in usual care arm; Panel B) Components of total energy intake from EN, PN and non-study PN in intervention arm

Panel A)

EN: Enteral nutrition; PN: Parenteral nutrition

| Day | 1 | 2 | 3 | 4 | 5 | 6 | 7 |
| --- | --- | --- | --- | --- | --- | --- | --- |
| Usual care (n) | 48 | 48 | 46 | 44 | 42 | 41 | 38 |
| Proportion of energy requirement provided in total | 24% | 60% | 71% | 75% | 80% | 72% | 71% |

Panel B)

EN: Enteral *nutrition*; PN: Parenteral nutrition

| Day | 1 | 2 | 3 | 4 | 5 | 6 | 7 |
| --- | --- | --- | --- | --- | --- | --- | --- |
| Intervention (n) | 51 | 51 | 48 | 47 | 43 | 43 | 36 |
| Proportion of energy requirement provided in total | 56% | 117% | 104% | 98% | 105% | 88% | 91% |

# Protocol deviations during study period

| **Deviation explanation** | **Times occurred during study** |
| --- | --- |
| Patient randomised but not eligible | 3 (2 intervention arm and 1 usual care) |
| Study PN not given when indicated (Supp PN group only) | 10 |
| Other types | 11 |
| Study PN run at the incorrect rate i.e. run at 10kcal/kg when it should have been 20kcal/kg | 0 |

# Adverse events

| **Event explanation** | **Times occurred during study** | **Related to the study** |
| --- | --- | --- |
| Medically unstable patient with PEA arrest | 1 (Intervention arm) | Unrelated |
| Persistent hyperglycaemia | 1 (Intervention arm) | Possibly related |
